# The effect of eugenol on the cariogenic properties of *Streptococcus mutans* and dental caries development in rats

**DOI:** 10.3892/etm.2013.1066

**Published:** 2013-04-17

**Authors:** JING-SHU XU, YAO LI, XUE CAO, YUN CUI

**Affiliations:** Department of Oral Medicine, Kunhua Hospital, The First People’s Hospital of Yunnan, Kunming, Yunnan 650032, P.R. China

**Keywords:** dental caries, *Streptococcus mutans*, acid production, adherence, glucosyltransferase, eugenol

## Abstract

Eugenol has been widely used in medicine due to its antibacterial, anti-inflammatory, antioxidant, anticancer and analgesic properties. The present study was designed to investigate the effects of eugenol on the cariogenic properties of *Streptococcus mutans* and dental caries development in rats. Eugenol demonstrated significant inhibitory effects against acid production by *S. mutans*. The synthesis of water-insoluble glucans by glucosyltransferases was reduced by eugenol. Eugenol also markedly suppressed the adherence of *S. mutans* to saliva-coated hydroxyapatite beads. Furthermore, topical application of eugenol reduced the incidence and severity of carious lesions in rats. These results suggest that the natural compound eugenol may be a useful therapeutic agent for dental caries.

## Introduction

Dental caries is a prevalent oral infectious disease, which is associated with various pathogenic microorganisms, including *Streptococcus mutans* and *Streptococcus sobrinus. S. mutans* is considered a crucial pathogen in the pathogenesis of dental caries (1). *S. mutans* produces glucosyltransferases (Gtfs) and synthesizes glucans from sucrose. Glucans are critical for bacterial accumulation on the tooth surface and the formation of cariogenic biofilms (2). Furthermore, *S. mutans* survive at low pH values and generate acids that result in the demineralization of tooth enamel, thereby initiating dental caries (3). Therefore, it has been proposed that disruption of the ability of *S. mutans* to form acids and glucans is an effective therapeutic approach for the treatment of dental caries.

Eugenol (4-allyl-2-methoxyphenol) is an aromatic molecule found in essential oils and various plants, including cloves, bay leaves and cinnamon leaves. Numerous studies have reported that eugenol possesses antibacterial, antiviral, antioxidant, anti-inflammatory and analgesic effects (4–6). Eugenol has been widely used in dentistry to treat toothache and pulpitis (7). A previous study indicated that eugenol may be an ideal natural agent for use in oral care products. Eugenol inhibits the growth and insoluble and soluble glucan synthesis of *S. sobrinus* (8). However, the therapeutic effect of eugenol against dental caries has not been thoroughly evaluated. Therefore, the aims of this study were: i) to examine the effects of eugenol on the adherence, acid production and insoluble glucan synthesis of *S. mutans in vitro* and ii) to determine the inhibitory effects of eugenol on caries development *in vivo*.

## Materials and methods

### Reagents

Eugenol and sodium fluoride were purchased from Sigma (St. Louis, MO, USA). Hydroxyapatite beads were obtained from Bio-Rad (Hercules, CA, USA). All other chemicals were of analytical grade and commercially available.

### Measurement of acid production by S. mutans 25175

Acid production was performed as described previously with slight modifications (9). Briefly, eugenol was added to 0.95 ml phenol red broth containing 1% glucose. Then, the mixture was inoculated with 0.05 ml *S. mutans* 25175 seed culture. After incubation at 37°C for 24 h, the pH values of the cultures were measured using a pH meter.

### Measurement of water-insoluble glucan synthesis by Gtfs

*S. mutans* 25175 was cultured at 37°C for 24 h in tryptic soy broth. The culture supernatant was salted out with solid ammonium sulfate to 70% saturation and then agitated at 4°C for 1 h. After centrifugation at 13,500 × g for 20 min, the precipitate was dialyzed against 10 mM potassium phosphate buffer (pH 6.0). The crude Gtfs preparation was stored at −80°C until further analysis.

Reaction mixtures consisting of 0.025 ml crude Gtfs preparation and 0.175 ml eugenol (final concentration, 0, 4, 8 and 16 mg/ml) in 0.8 ml 0.0625 M potassium phosphate buffer containing 12.5 *μ*g/l sucrose and 0.25 *μ*g/l sodium azide were incubated at 37°C for 18 h. The water-insoluble glucan was sedimented and washed with distilled water and then ultrasonicated for 6 sec. The absorbance was examined at 550 nm against a blank control.

### Bacterial adherence assay

The bacterial adherence assay was performed as described previously (12). Briefly, *S. mutans* 25175 was diluted in tryptic soy broth at a density of 10^8^ colony-forming units (CFU) per milliliter. Human saliva was collected from an adult donor and clarified by centrifugation. Hydroxyapatite beads were treated with clarified human saliva and rotated at 7 × g for 1 h at room temperature. The saliva-coated hydroxyapatite beads (S-HAs) were washed three times with 0.01 M potassium phosphate buffer (pH 7.0). Bacterial suspensions with or without various concentrations of added eugenol were incubated with the S-HAs at 37°C for 90 min and then the S-HAs were washed three times with potassium phosphate buffer. The detached cells on S-HAs were dispersed, diluted and spread on Mitis Salivarius agar plates containing bacitracin (3.2 mg/ml). The number of bacterial colonies was counted on each plate after incubation at 37°C for 48 h and the CFU were then calculated.

### Animal experiments

The animal experiments were performed as described previously (10,11). Briefly, specific pathogen-free male Wistar rats (aged 19 days; Kunming Medical University, Kunming, China) were infected daily for five consecutive days with a growing culture of *S. mutans* 25175. Rats aged 25 days were randomly divided into three groups (n=15) and their teeth were treated topically using a camel hair brush twice daily for 5 weeks, as follows: i) vehicle control [15% ethanol containing 1% dimethylsulfoxide (DMSO)], ii) 16 mg/ml eugenol and iii) 250 ppm fluoride. The rats were provided with cariogenic diet 2000 and 5% sucrose water *ad libitum*. At the end of the 5-week experimental period, the rats were deeply anesthetized and sacrificed. The lower left jaw was collected aseptically, immerged in 5.0 ml sterile saline solution and sonicated. The suspension was plated on blood agar and on Mitis Salivarius agar plus streptomycin to respectively estimate the number of total cultivable microorganisms and *S. mutans* populations. The smooth-surface and sulcal caries and their severities (Ds, dentin exposed; Dm, 3/4 of the dentin affected; Dx, whole dentin affected) were evaluated by means of Larson’s modification of Keyes’s system (11,12). The caries score was determined blindly with respect to the groups. All procedures were performed in accordance with guidelines set for the use of experimental animals by the Committee on Animal Care and Use of Kunhua Hospital (Kunming, China).

### Statistical analysis

All values are expressed as the mean ± standard error of the mean (SEM). The data were analyzed by analysis of variance (ANOVA) followed by Tukey-Kramer multiple comparisons test using SPSS software (SPSS, Inc., Chicago, IL, USA). P<0.05 was considered to indicate a statistically significant difference.

## Results

### In vitro effect of eugenol on the cariogenic properties of S. mutans

**Figure 1:**
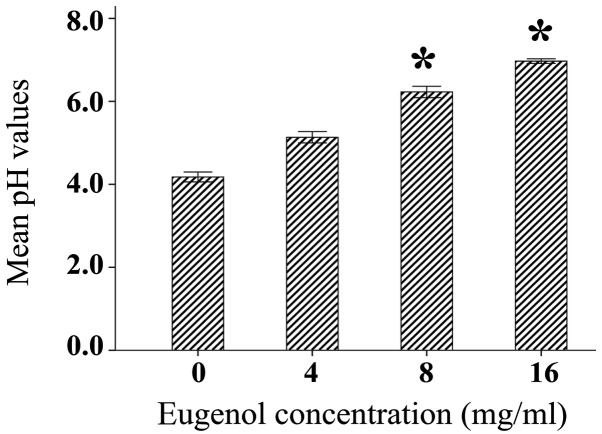
Effects of eugenol on acid production by *Streptococcus mutans* 25175. The assay was performed in triplicate and data are presented as mean ± standard error of the mean (SEM). ^*^P<0.05, compared with the control.

In this study, we firstly investigated the inhibitory effect of eugenol on acid production by *S. mutans* 25175. The cells were treated with various concentrations of eugenol and the pH was then measured. As shown in Fig. 1, the acid production was significantly suppressed by eugenol compared with the control group.

**Figure 2:**
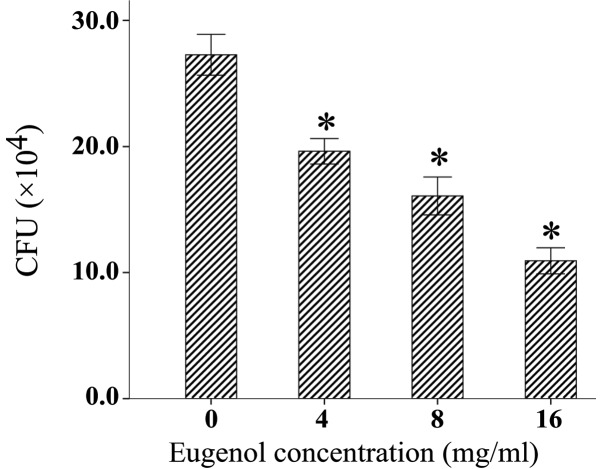
Effect of eugenol on the cell adherence of *Streptococcus mutans* 25175. The number of colony forming units (CFU) of *S. mutans* adhering to saliva-coated hydroxyapatite beads at various concentrations of eugenol was measured. The assay was performed in triplicate and data are presented as mean ± standard error of the mean (SEM). ^*^P<0.05, compared with the control.

To assess the effects of eugenol on the adherence of *S. mutans* 25175, a bacterial adherence assay was performed. As shown in Fig. 2, eugenol significantly inhibited the adherence of *S. mutans* to S-HAs in a concentration-dependent manner.

**Figure 3:**
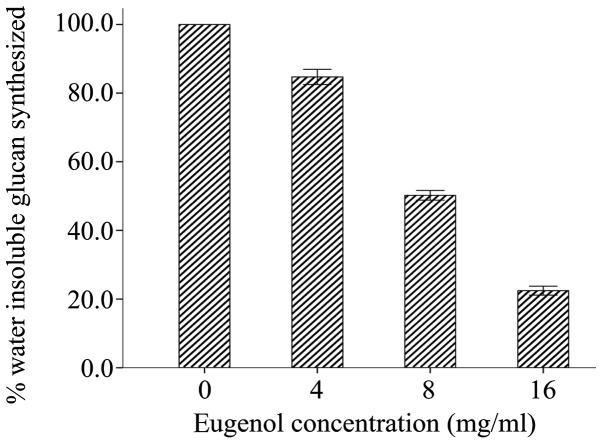
Effect of eugenol on insoluble glucan synthesis by glucosyltransferases (Gtfs) of *Streptococcus mutans* 25175. The amount (%) of insoluble glucan produced by various concentrations of eugenol was determined relative to the control. The assay was performed in triplicate and data are presented as mean ± standard error of the mean (SEM). ^*^P<0.05, compared with the control.

We also examined whether eugenol suppresses the synthesis of water-insoluble glucans by crude Gtfs. As shown in Fig. 3, a significant reduction in water-insoluble glucan synthesis by crude Gtfs from *S. mutans* was observed at concentrations of 4–16 mg/ml eugenol.

### Inhibitory effects of eugenol on dental caries development in rats

In the animal experiment, the rats remained in good health and gained weight during the 5 weeks of the study. No significant differences in weight gain were identified among the groups (P>0.05, data not shown).

The effects of eugenol on the total cultivable flora, *S. mutans* viable populations and percentage of *S. mutans* recovered from the rat jaws (as calculated from total cultivable flora, *S. mutans* population) are shown in Table I. The eugenol-treated group exhibited significantly lower total flora counts compared with the vehicle control. However, the number of CFUs and percentage of *S. mutans* in the biofilms of the rats treated with eugenol did not differ significantly from those of the vehicle control.

Tables II and III show the incidence and severity of smooth-surface and sulcal caries. In the present study, 250 ppm fluoride was used as a positive control. The 250 ppm fluoride treatment produced the lowest scores for incidence and severity of smooth-surface and sulcal caries. Eugenol treatment significantly reduced the incidence of smooth-surface and sulcal caries compared with the vehicle control. Furthermore, the severity scores of smooth-surface and sulcal caries were significantly lower in the eugenol-treated group compared with those in the vehicle control group.

## Discussion

Despite advances in the development and improvement of anti-caries chemotherapy, conventional therapeutic strategies often fall short of the goal of controlling dental caries progression. The use of natural products has been reported to be one of the most successful strategies for the discovery of new medicines (11,13). Previous studies have shown that natural products are promising candidates for new anticariogenic substances (9,13). The present study demonstrated that eugenol, a naturally occurring agent, interferes with cariogenic factors of *S. mutans*, including acid production, adherence and water-insoluble glucan synthesis *in vitro* and inhibited the development of dental caries in rats.

Acid production is an important dental caries-related factor of *S. mutans* (14). In dental biofilms, *S. mutans* metabolize sugars to produce organic acids, including lactic acid, propionic acid and butyric acid, which may demineralize the tooth surface and thereby induce dental caries (3). In the present study, eugenol significantly inhibited the reduction of pH induced by *S. mutans*. These results suggest that eugenol may reduce acid production by *S. mutans*.

The adherence of *S. mutans* to the tooth surface is one of the most important steps for dental plaque formation (9,15). Disruption of the ability of *S. mutans* to adhere to the surface of the tooth is considered an important therapeutic approach for the prevention of plaque formation. Therefore, we investigated the effect of eugenol on the adhesion of *S. mutans* to S-HAs. At concentrations of 4–16 mg/ml, eugenol significantly reduces the adherence of *S. mutans* to S-HAs. These data suggest that eugenol may be a novel substance capable of modulating the activity of this important dental caries-related factor.

The synthesis of extracellular polysaccharides, including water-insoluble glucans, is one of the most important virulent properties of *S. mutans* (16). Water-insoluble glucans promote the adhesive interactions of bacteria with the tooth surface and contribute to the formation of dental biofilms (17). Accordingly, we examined whether eugenol inhibits the synthesis of water-insoluble glucans by crude Gtfs. The results showed that the formation of water-insoluble glucans by *S. mutans* was significantly suppressed in the presence of eugenol. Our results correlate well with other reports that eugenol suppresses the insoluble and soluble glucan synthesis of *S. sobrinus* (8,18). *S. mutans* synthesizes glucans from sucrose by the action of Gtfs. There are at least three types of Gtfs in *S. mutans*: Gtf B, Gtf C and Gtf D. It has been reported that Gtf B and C are important for the synthesis of insoluble glucans by *S. mutans* (19,20). Gtf B synthesizes primarily insoluble glucans, whereas Gtf C synthesizes insoluble and soluble glucans. Although we identified that eugenol inhibits water-insoluble glucan synthesis, it was not confirmed whether eugenol inhibits Gtf B and/or Gtf C. This requires further investigation.

The inhibitory effects of eugenol on the acid production, adherence and water-insoluble glucan synthesis activities of *S. mutans* may be beneficial for the prevention of caries development *in vivo*. Therefore, we examined the anti-caries activity of eugenol using a rat model of dental caries. Topical application of eugenol reduces the incidence and severity of carious lesions in rats without affecting the number and percentage of *S. mutans* in the biofilms. In smooth-surface caries, eugenol effectively reduced the number and severity of caries, with results similar to those of 250 ppm fluoride (the positive control). However, the inhibitory effects of eugenol in sulcal caries were not as effective as those exhibited by 250 ppm fluoride. These results suggest that eugenol is an effective agent for the inhibition of dental caries development *in vivo*. Natural products that inhibit cariogenic properties of *S. mutans* may be used to control dental caries or even to enhance the cariostatic effect of other recognized agents, including fluoride. Therefore, further studies are required to investigate the possible additive or synergistic anticariogenic effects of eugenol and fluoride.

In conclusion, our data demonstrated that eugenol significantly attenuates the acid production, adherence and water-insoluble glucan synthesis activities of *S. mutans* and suppresses dental caries development in rats. These results suggest that eugenol is a promising naturally occurring agent for the treatment of dental caries.

## Figures and Tables

**Table I:** Effects of eugenol on oral microbiota in rats after a 5-week experiment.

Group	Total microorganisms (×10^4^ CFU/ml)	*Streptococcus Mutans* 25175 (×10^4^ CFU/ml)	*Streptococcus Mutans* 25175 (%)
Vehicle	4.3^a^ (1.6)	3.0^a^ (1.8)	69.9^a^ (20.1)
Eugenol (16 mg/ml)	2.4^b^ (0.6)	1.6^a^ (0.4)	67.7^a^ (18.4)
250 ppm fluoride	2.2^b^ (0.5)	1.7^a^ (0.3)	77.3^a^ (16.2)

Statistical analyses for all pairs was performed using Tukey-Kramer multiple comparisons test (n=15). Fluoride was used as a positive control. Values followed by different lower case letters (vertical) are significantly different from each other (P<0.05). CFU, colony forming units.

**Table II:** Effects of treatments on dental caries development (smooth surface and severity) in rats.

Group	Smooth surface total	Severity		
		Ds	Dm	Dx
Vehicle	63.1^a^ (5.7)	41.6^a^ (6.7)	18.7^a^ (4.3)	7.4^a^ (5.1)
Eugenol (16 mg/ml)	39.2^b^ (5.2)	22.1^b^ (4.8)	4.8^b^ (5.8)	2.0^b^ (0.8)
250 ppm fluoride	23.2^c^ (3.1)	17.7^b^ (5.7)	0.7^b^ (0.5)	0.1^c^ (0.4)

Statistical analyses for all pairs was performed using Tukey-Kramer multiple comparisons test (n=15). Values followed by different lower case letters (vertical) are significantly different from each other (P<0.05). Ds, dentin exposed; Dm, 3/4 of the dentin affected; Dx, whole dentin affected.

**Table III:** Effects of treatments on dental caries development (sulcal surface and severity) in rats.

Group	Sulcal surface total	Severity		
		Ds	Dm	Dx
Vehicle	37.8^a^ (4.8)	26.3^a^ (4.5)	20.3^a^ (3.7)	13.4^a^ (2.2)
Eugenol (16 mg/ml)	28.5^b^ (3.8)	16.7^b^ (4.1)	9.2^b^ (3.6)	5.9^b^ (2.8)
250 ppm fluoride	18.9^c^ (3.1)	9.5^c^ (4.1)	2.9^c^ (1.4)	0.3^c^ (0.2)

Statistical analyses for all pairs was performed using Tukey-Kramer multiple comparisons test (n=15). Values followed by different lower case letters (vertical) are significantly different from each other (P<0.05). Ds, dentin exposed; DM, 3/4 of the dentin affected; Dx, whole dentin affected.
